# Supplementary material for: Integration of single-cell transcriptomes and chromatin landscapes reveals regulatory programs driving pharyngeal organ development
Source: Nat Commun. 2022 Jan 24;13:457. doi: 10.1038/s41467-022-28067-4 (PMC8786836; doi:10.1038/s41467-022-28067-4)
Supplement: Supplementary file 1 — Supplementary Information [file 41467_2022_28067_MOESM1_ESM.pdf]

Supplementary Table 1. Cell numbers by embryonic day per single-cell RNA cluster.

	Cell number by embryonic day			
Louvain cluster	E9.5	E10.5	E11.5	E12.5
0	17	2220	3095	55
1	3833	85	14	1
2	5	2152	1077	28
3	8	2408	738	2
4	0	0	42	2885
5	10	1359	1484	20
6	0	0	84	2717
7	0	33	1327	1301
8	10	643	1615	135
9	0	63	1899	131
10	502	892	663	3
11	3	355	1408	151
12	2	560	883	372
13	1741	25	2	0
14	1672	57	5	1
15	5	759	789	29
16	1388	143	24	2
17	1352	31	0	0
18	0	0	221	1006
19	9	711	411	84
20	0	0	16	1108
21	1050	38	10	0
22	26	296	301	313
23	890	12	0	0
24	819	40	0	0
25	0	0	3	521
26	0	9	247	194
27	3	229	135	27

Supplementary Table 1. Cell numbers by embryonic day per single-cell RNA cluster. Total number of cells by embryonic day per cluster in the single-cell transcriptomic data set (See Supplementary Fig. 3 for UMAP with cluster labels).

Supplementary Figure 1

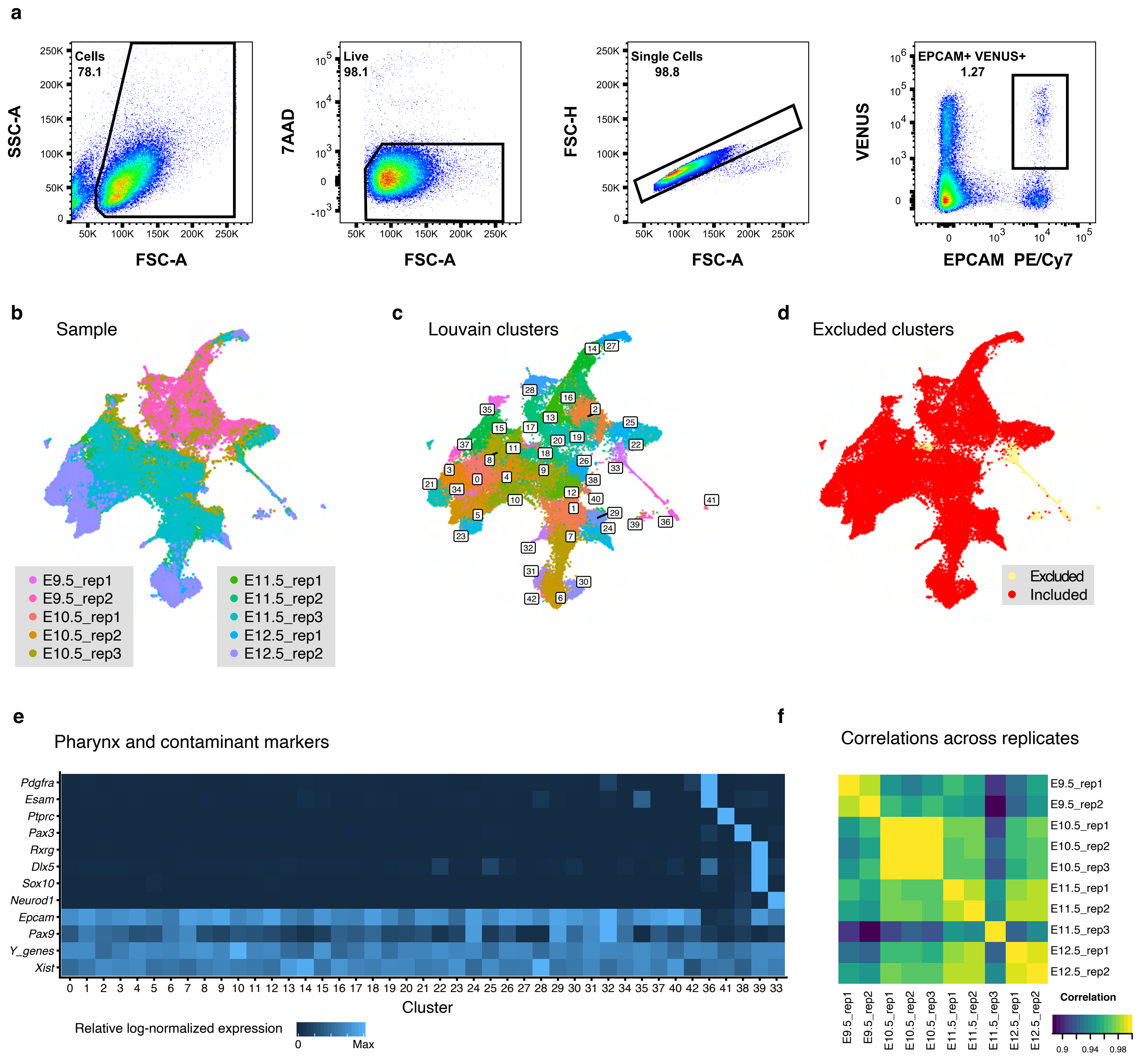

Supplementary Figure 1. Pharyngeal endoderm single-cell transcriptomic data cleaning.

a) Representative gating strategy for FACS purification of VENUS/Epcam pharyngeal endoderm cells.

b-d) UMAP visualization of all pharyngeal endoderm cells. Each dot is one cell in the global transcriptomic space. Color marks sample (**b**), Louvain clusters (**c**), or excluded clusters (**d**).

e) Expression of select contaminant markers and quality-control measures over all clusters.

f) Pearson correlations of per-transcript raw counts across samples. In each sample, all transcripts were counted after CellRanger's automated empty-droplet exclusion but prior to the cell exclusion in (**d**).

Supplementary Figure 2

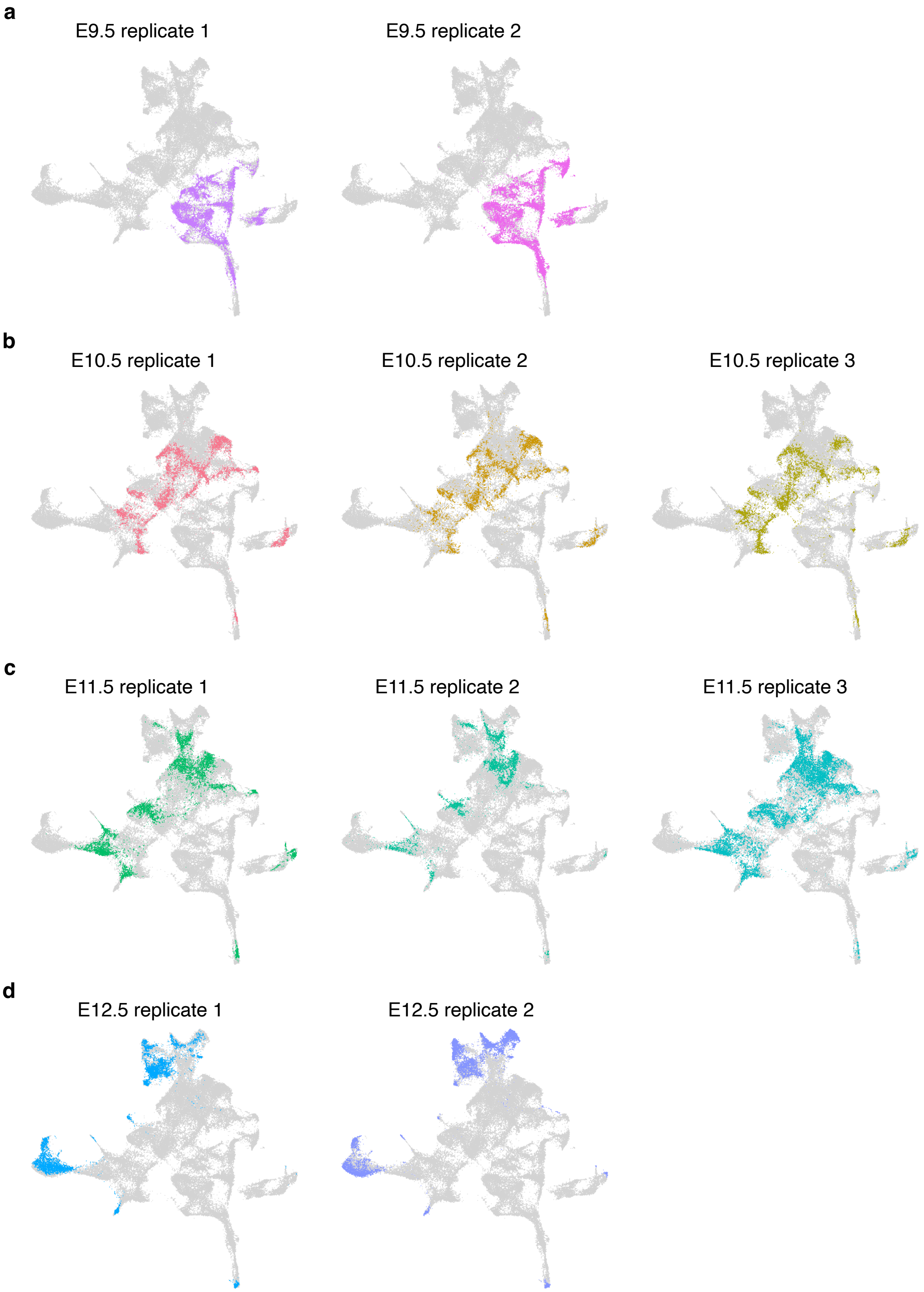

Supplementary Figure 2. Pharyngeal endoderm single-cell transcriptomic replicate composition.

a-d) UMAP visualization of individual replica by embryonic day: E9.5 (**a**), E10.5 (**b**), E11.5 (**c**), E12.5 (**d**).

Supplementary Figure 3

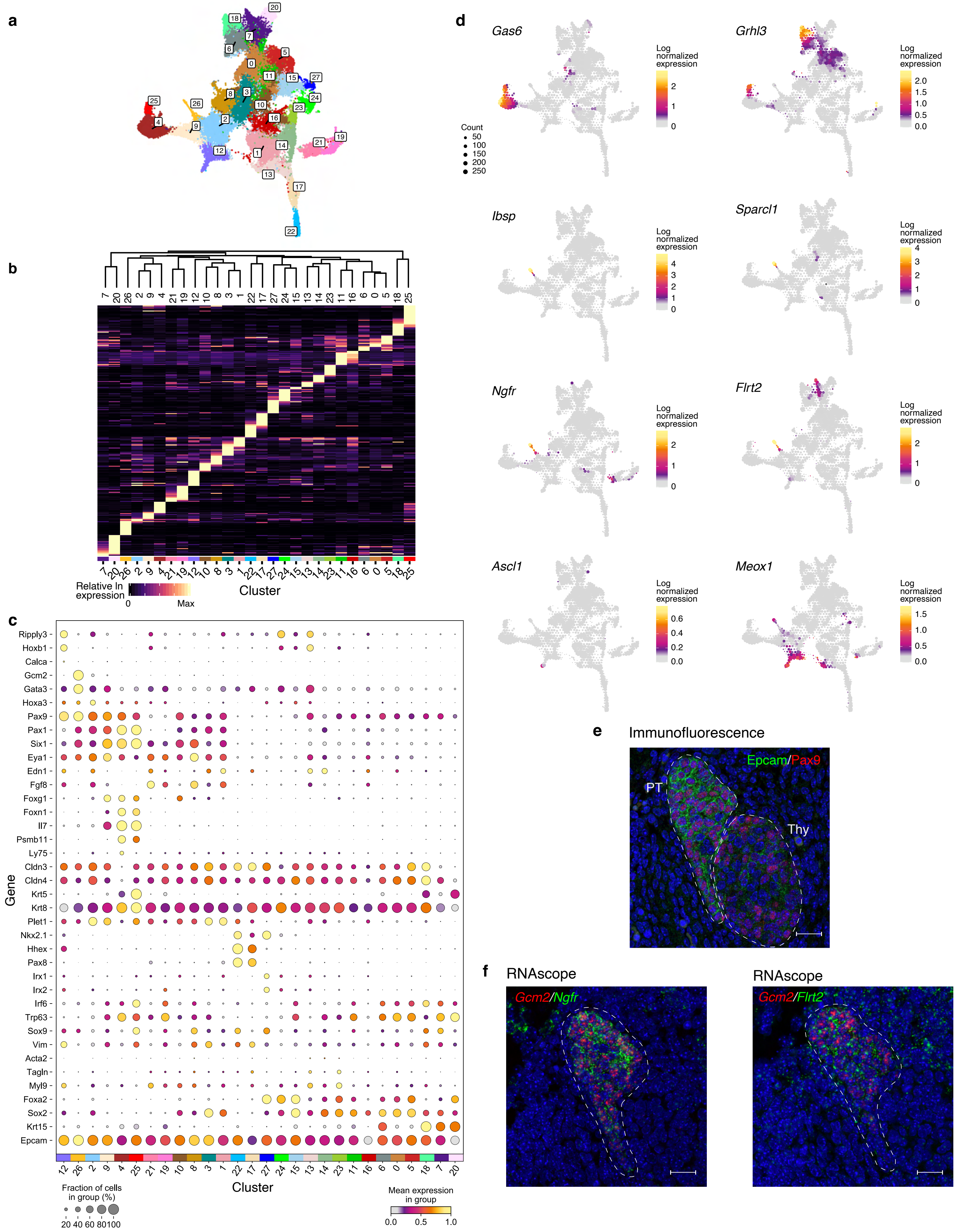

Supplementary Figure 3. In-depth heterogeneity analysis of pharyngeal endoderm.

a) UMAP visualization displaying Louvain cluster identities. Each dot represents one cell positioned according to the global transcriptomic space, with color signifying cluster assignment.

b) Top differentially expressed marker genes for all clusters, ranked by log2-fold change.

c) Dot plot displaying expression patterns of curated tissue-specific and pharyngeal endoderm genes. The size of the dot indicates the fraction of cells per cluster expressing a given gene, and the color indicates normalized mean expression per cluster.

d) UMAP visualization displaying expression of genes verified by RNAscope. Cells are binned by UMAP coordinates, and one dot represents one bin, with size proportional to the number of cells and color signifying average log2-normalized expression.

e) Immunofluorescence stain of Epcam and Pax9 expression on E12.5 tissue sample (n = 2). Scale bars represent 20 μm.

f) RNAscope *in situ* hybridization of parathyroid-specific marker genes (data-driven: *Ngfr, Flrt2*; known: *Gcm2*; n = 2). Scale bars represent 20 μm.

Supplementary Figure 4

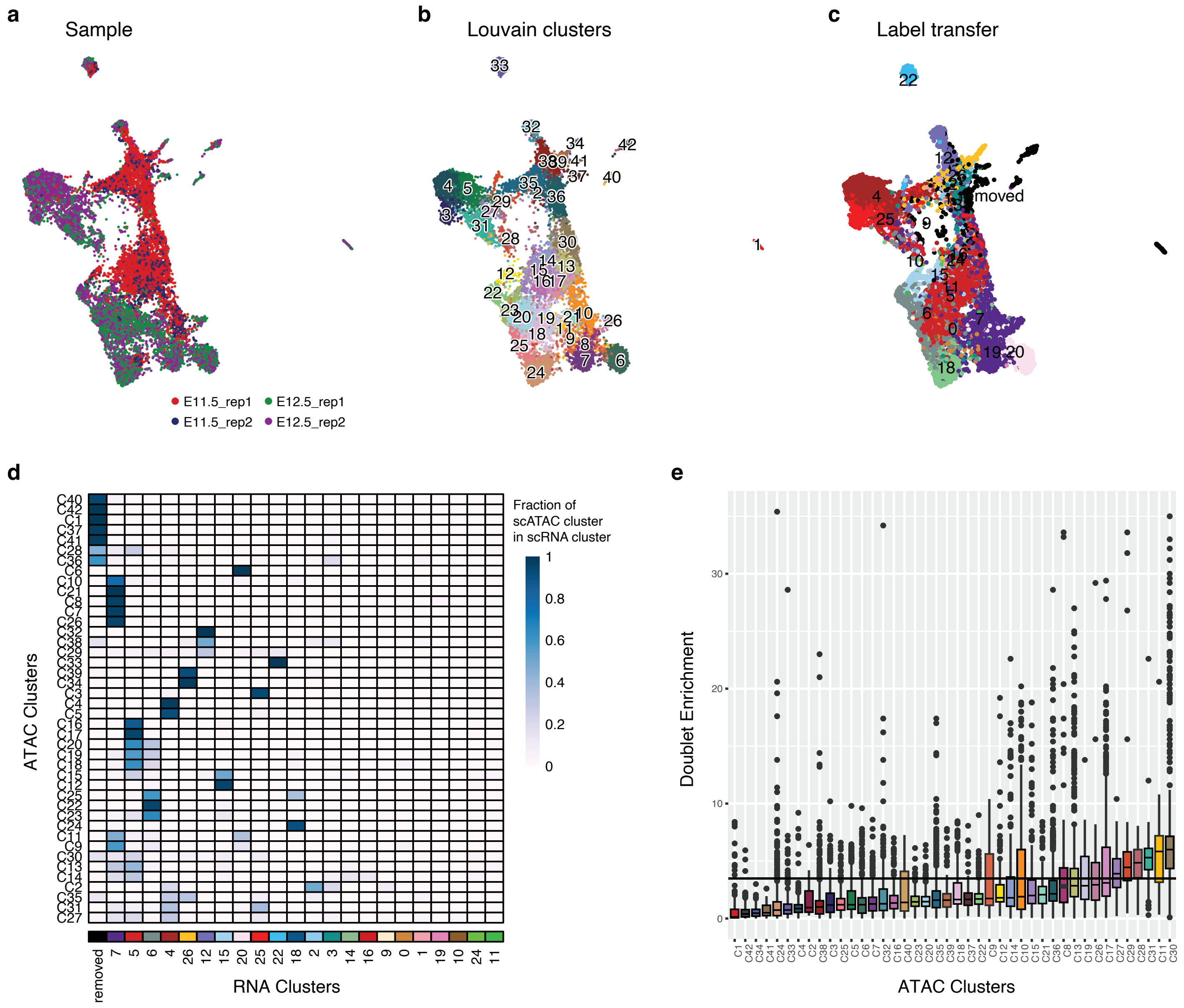

Supplementary Figure 4. Pharynx single-cell ATAC atlas data cleaning.

a-c) UMAP visualization of atlas single-cell ATAC dataset after removal of low-quality cells (Frags < 3000, TSS enrichment < 4). Color marks sample (**a**), Louvain clusters (**b**), and labels transferred from the pre-filtered scRNA atlas (Supplementary Fig. 1d) with cluster assignment from the transcriptomic atlas (Supplementary Fig. 3a) (**c**). Cells excluded in the transcriptomic atlas are labeled “removed”. One dot represents one cell in the global chromatin landscape space.

d) Heatmap showing the fraction of cells in each cluster mapping to the transcriptomic atlas clusters from Supplementary Fig. 3a and the cells excluded from the atlas in Supplementary Fig. 1d (labeled “removed”).

e) Boxplot showing the doublet enrichment for n=13,396 cells across the 42 scATAC louvain clusters. The center line denotes the median; the box limits denote the upper and lower quartiles; the top and bottom whiskers denote the region up to 1.5 times the inter-quartile range above the upper and below the lower quartiles respectively; and the points outside this region denote outliers. Doublet enrichment was computed independently for each of the 4 scATAC samples using the ArchR package. Clusters are ordered by increasing median doublet enrichment. The threshold (1 standard deviation above the mean on the distribution of cluster-median doublet enrichment) to filter clusters enriched in doublets is indicated by a black horizontal line.

Supplementary Figure 5

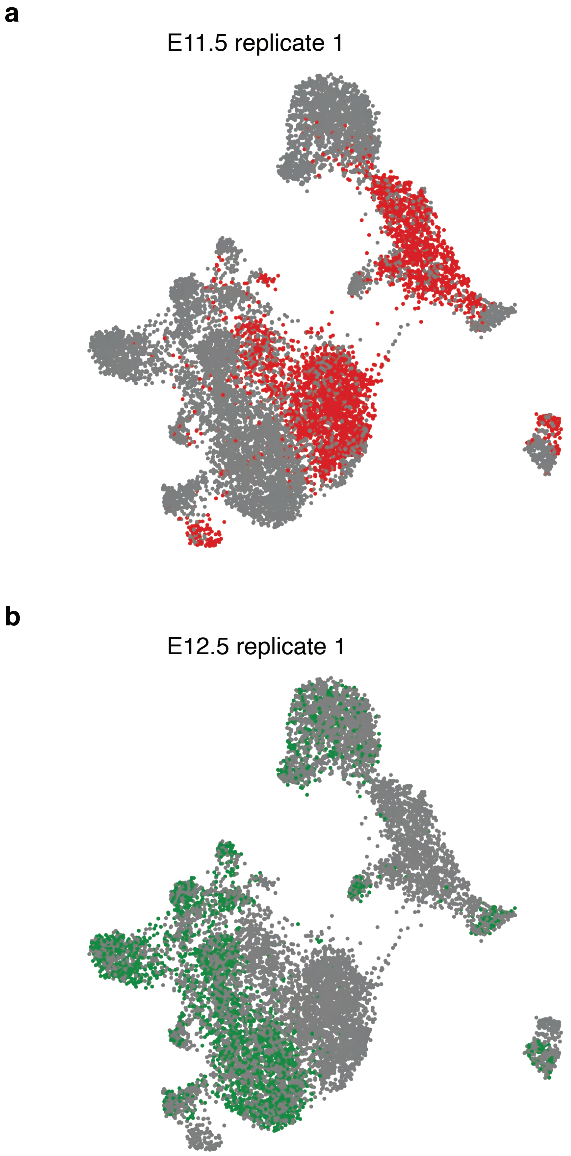

Supplementary Figure 5. Pharyngeal endoderm single-cell ATAC replicate composition.

a, b) UMAP visualization of individual replica by embryonic day: E11.5 (**a**) and E12.5 (**b**).

Supplementary Figure 6

a

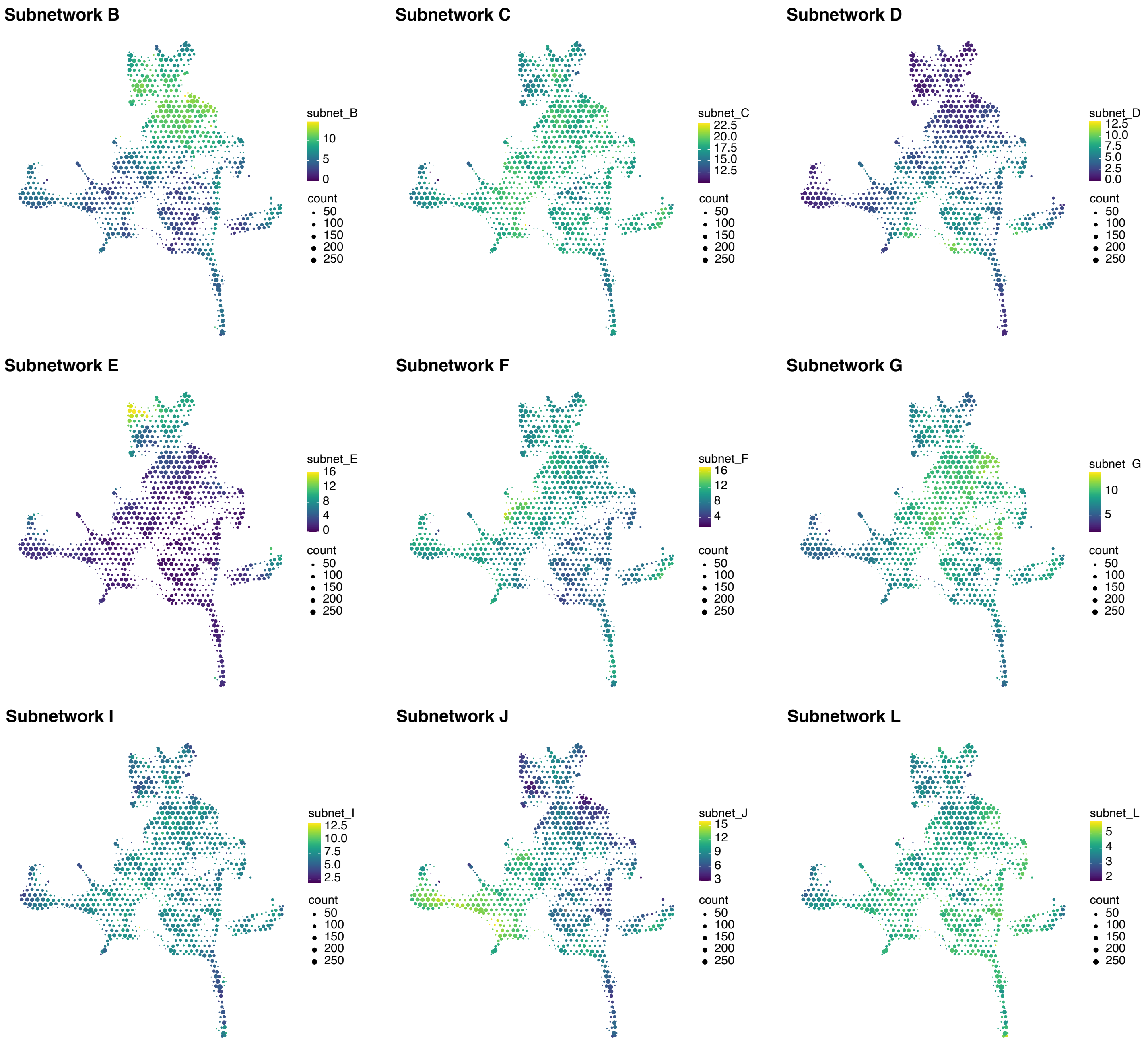

Supplementary Figure 6. GENIE3 subnetwork UMAP visualizations.

a) UMAPs displaying mean scRNA expression of subnetwork specific genes. Cells are binned by UMAP coordinate with size proportional to the number of cells and color signifying average log2-normalized expression.

Supplementary Figure 7

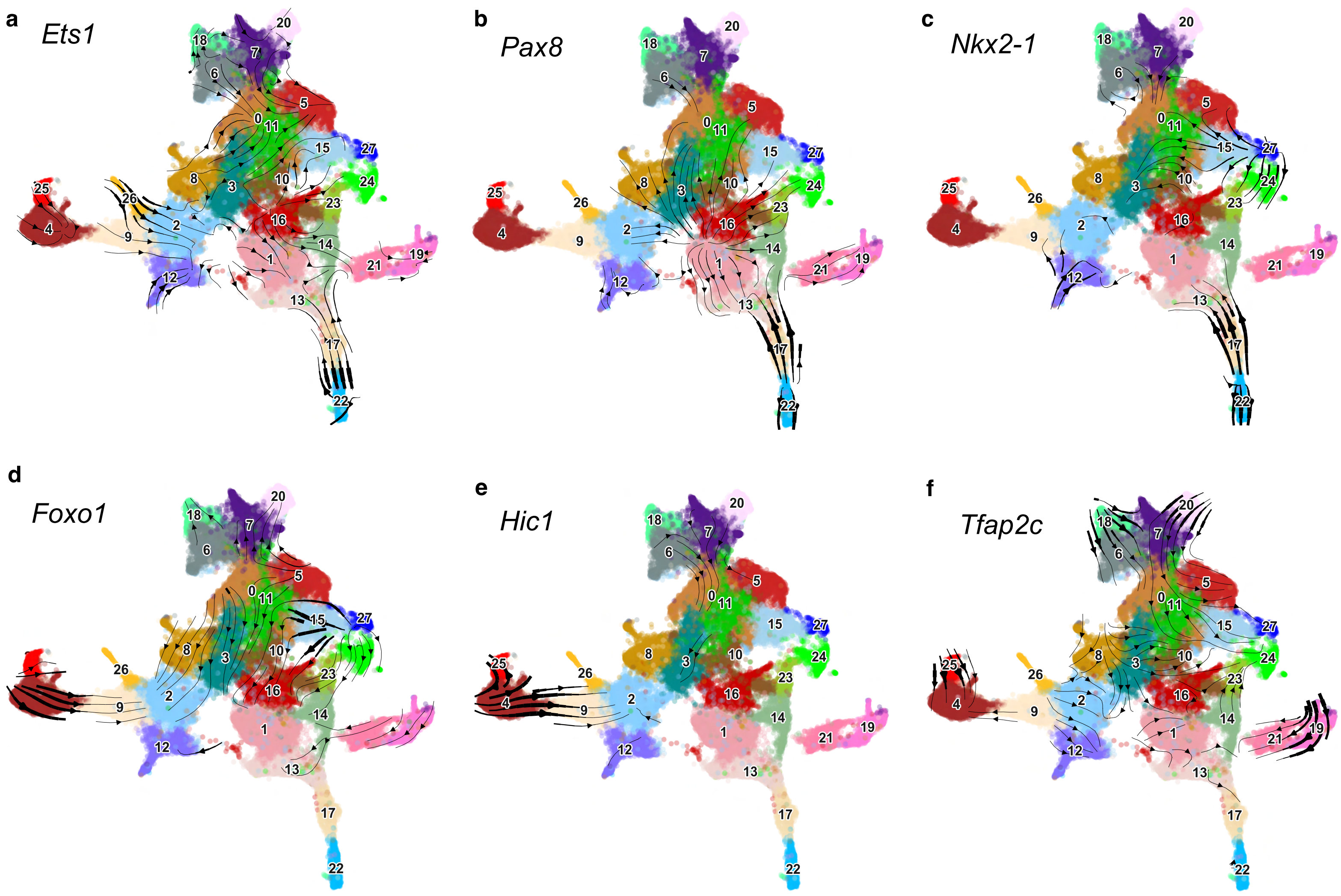

Supplementary Figure 7. Simulated genetic knockouts of predicted regulators of various organ domains.

a-f) Visualization of an inducible knockouts as simulated by CellOracle for the following genes/lineages: *Ets1* (**a,** parathyroid), *Pax8* (**b**, thyroid), *Nkx2.1* (**c**, thyroid, respiratory, UBB), *Foxo1* (**d**, thymus), *Hic1* (**e**, thymus), and *Tfap2c* (**f**, thymus). Changes in gene expression between the simulated knockout and atlas cells are displayed as velocity streams on the UMAP embedding using scVelo.

Supplementary Figure 8

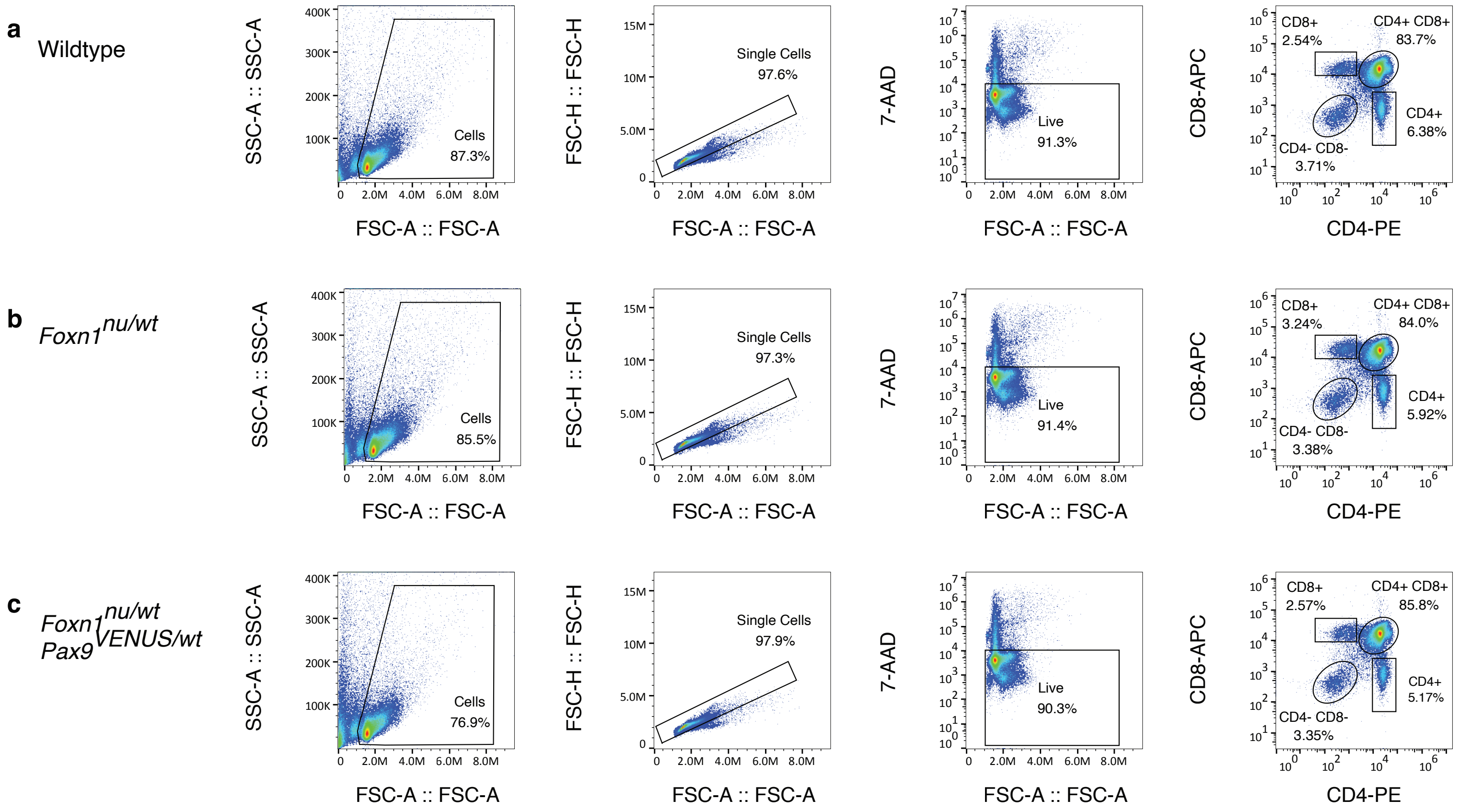

Supplementary Figure 8. Functional analysis of *Foxn1*$^{nu/wt}$*Pax9*$^{VENUS/wt}$ heterozygote.

a-c) Thymocytes isolated from 5-week postnatal thymi were analyzed via flow cytometry. Visualization of cells (left panel), single cells (2nd panel), and live cells (3rd panel) shows similar percentages of CD4+, CD8+, double negative, and double positive thymocytes (4th panel). Each sample represents one animal of a unique genotype: wildtype (**a**), *Foxn1*$^{nu/wt}$ (**b**), and Foxn1$^{nu/wt}$/Pax9$^{VENUS/wt}$ (**c**).

Supplementary Figure 9

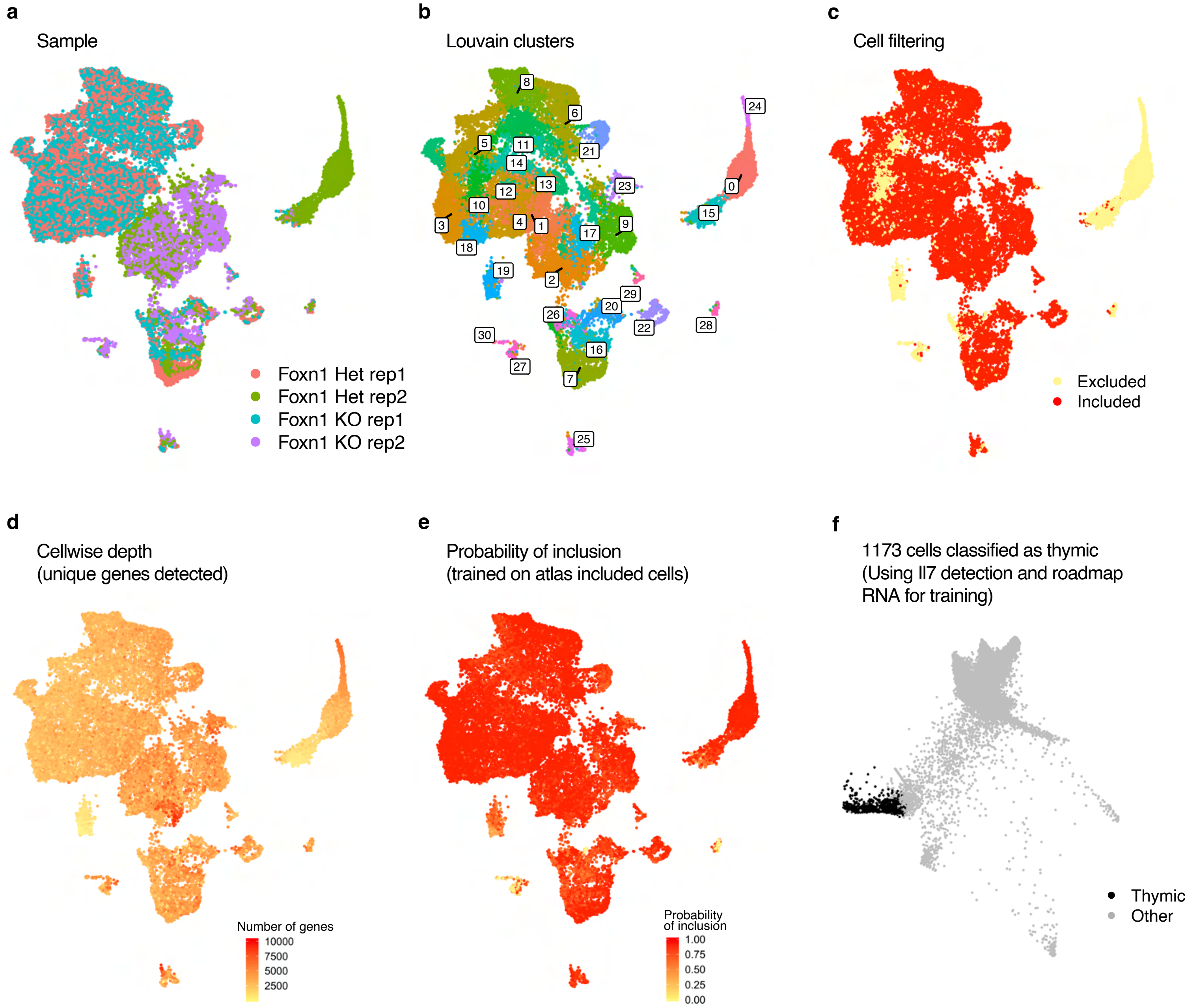

Supplementary Figure 9. Foxn1 experiment RNA data cleaning and cell type annotation.

a-e) UMAP representation of Foxn1 experiment cells. Each dot is one cell, and color marks sample (**a**), Louvain clusters (**b**), cells removed (**c**), genes detected (**d**), and contamination probability (**e**). Inclusion probability is assessed via a binary nearest-neighbors classifier using the atlas included cells as positive training examples.

f) Isolation of thymic cells using Il7 detection (>0 counts) in combination with a classifier based on the atlas scRNA.

Supplementary Figure 10

a

Foxn1 heterozygous replicate 1 | Foxn1 heterozygous replicate 2

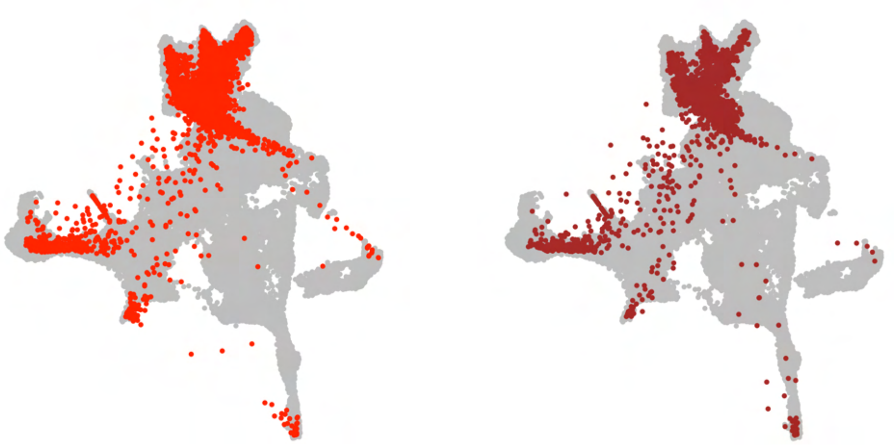

b

Foxn1 knockout replicate 1 | Foxn1 knockout replicate 2

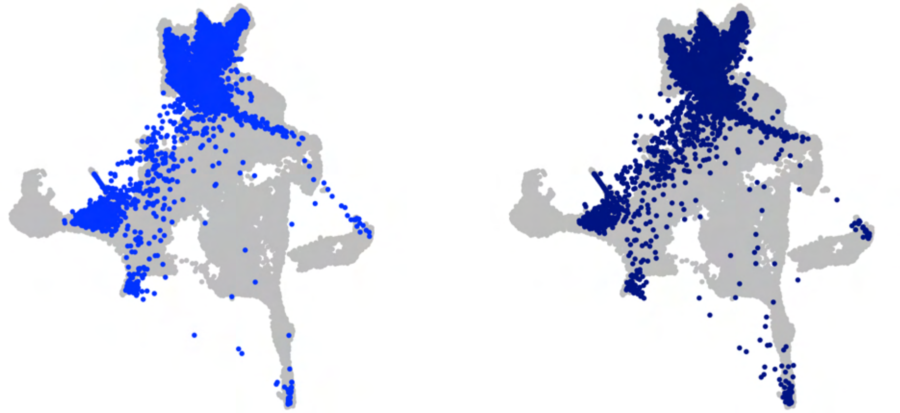

Supplementary Figure 10. Foxn1 validation single-cell RNA replicate composition.

a, b) UMAP visualization of individual replica by genotype: *Foxn1*$^{nu/wt}$ (**a**) and *Foxn1*$^{nu/nu}$ (**b**).

Supplementary Figure 11

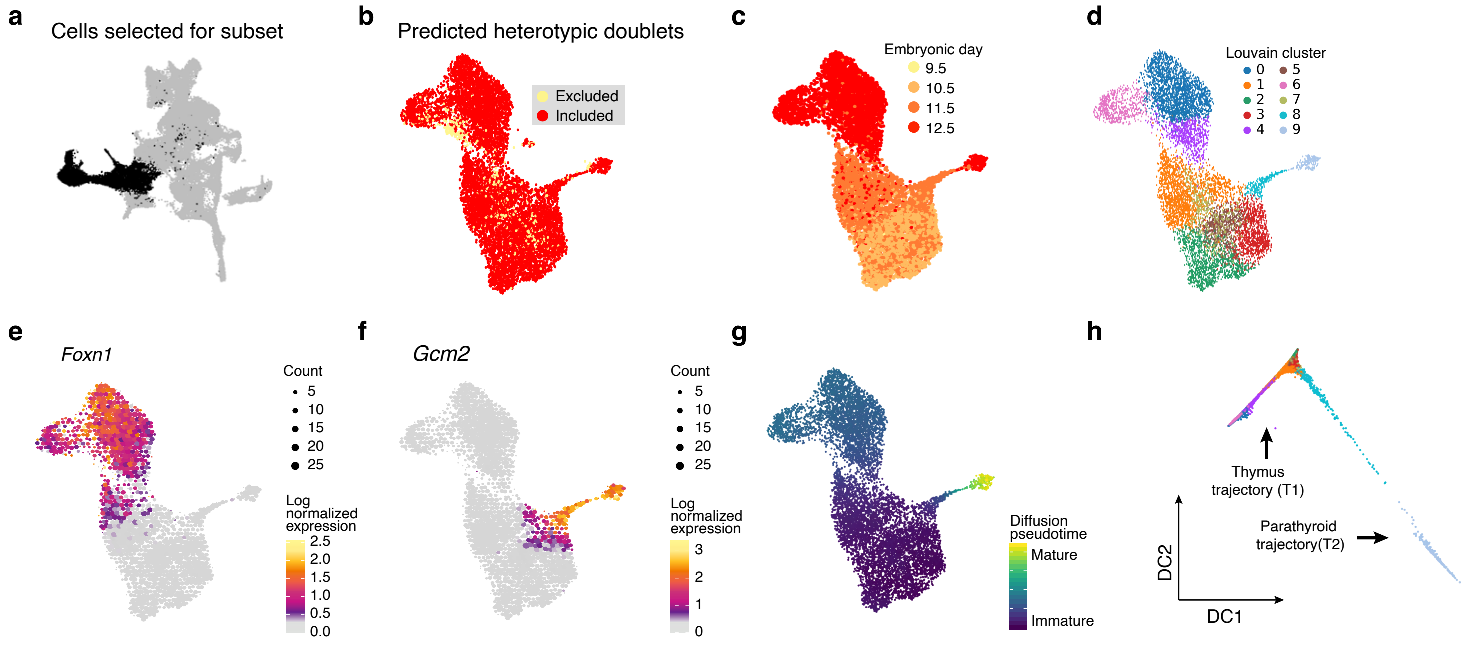

Supplementary Figure 11. Third pouch subset pseudotime and trajectory analysis.

a) Cells (*n* = 9,256 cells) from clusters 2, 9, 4, 25 and 26 of the scRNA atlas selected for subset analysis

b) UMAP visualization of likely heterotypic doublets as identified by doubletFinder.

c, d) UMAP visualization of third pouch subset (*n* = 8,717 cells) colored by embryonic day (**c**) and Louvain clusters (**d**). Each dot represents a single cell in the global transcriptomic space.

e, f) UMAP visualization of expression of select lineage-specific marker genes of the thymus (**e**) and the parathyroid (**f**). Cells are binned by UMAP position, and each dot represents one bin.

g) Diffusion pseudotime (DPT) ranking of third pouch subset cells.

h) Diffusion components 1 and 2 from DPT analysis. Colors represent unsupervised clustering results as in (**d**).
