# Supplementary material for: Integration of single-cell transcriptomes and chromatin landscapes reveals regulatory programs driving pharyngeal organ development
Source: Nat Commun. 2022 Jan 24;13:457. doi: 10.1038/s41467-022-28067-4 (PMC8786836; doi:10.1038/s41467-022-28067-4)
Supplement: Supplementary file 3 — Description of Additional Supplementary Files [file 41467_2022_28067_MOESM3_ESM.docx]

Description of Supplementary Datasets included in the manuscript ““Integration of single-cell transcriptomes and chromatin landscapes reveals regulatory programs driving pharyngeal organ development”.

**Supplementary Dataset 1. Top marker genes per cluster in the transcriptomic atlas.** Top differentially expressed genes corresponding to each cluster of the single-cell RNA data reported in Fig. 1c and Supplementary Fig. 3.

**Supplementary Dataset 2. Top 100 gene score markers per single-cell ATAC cluster.** Top differentially expressed gene scores as defined by ArchR corresponding to each cluster of the single-cell ATAC data reported in Fig. 2c.

**Supplementary Dataset 3. Differentially accessible peaks across single-cell ATAC clusters.** Differentially accessible peaks corresponding to each cluster of the single-cell ATAC data reported in Fig. 2c.

**Supplementary Dataset 4. Top regulators per GENIE3 subnetwork ranked by out-degree.** Transcription factor regulators of each GENIE3 subnetwork ranked by out-degree. Relevant to Fig. 4b-e and Supplementary Fig. 6.

**Supplementary Dataset 5. CellOracle network summary metrics per single-cell RNA cluster.** CellOracle network summary scores for each single-cell RNA cluster reported in Fig. 1c and Supplementary Fig. 3. Relevant to Fig. 4f,g.

**Supplementary Dataset 6. Differential gene expression between Foxn1 knockout and control.** Gene expression comparison between the Foxn1 knockout sample with the heterozygous control. Relevant to Fig. 6d,e.

**Supplementary Dataset 7. Comparison of changes between Foxn1 knockout and control to developmental changes in atlas counterpart cells.** Gene expression changes between the Foxn1 knockout and heterozygous control, and the E11.5 and E12.5 atlas counterpart cells. Relevant to Fig. 6c.

**Supplementary Dataset 8. EnrichR analysis of Foxn1 knockout versus control differentially expressed genes.** EnrichR terms compiled from several databases associated with genes differing between the Foxn1 knockout and heterozygous control. Relevant to Fig. 6d,e.

**Supplementary Dataset 9. Top genes per cluster in third pouch subset analysis.** Differential gene expression results of clusters in the third pouch subset, which was used to stage Foxn1 knockout and control cells along a pseudotime trajectory of the thymus. Relevant to Fig. 6a,b.
