# Supplementary material for: Integration of single-cell transcriptomes and chromatin landscapes reveals regulatory programs driving pharyngeal organ development
Source: Nat Commun. 2022 Jan 24;13:457. doi: 10.1038/s41467-022-28067-4 (PMC8786836; doi:10.1038/s41467-022-28067-4)
Supplement: Supplementary file 13 — Reporting Summary [file 41467_2022_28067_MOESM13_ESM.pdf]

nature portfolio

Corresponding author(s): René Maehr

Last updated by author(s): Nov 5, 2021

Reporting Summary

Nature Portfolio wishes to improve the reproducibility of the work that we publish. This form provides structure for consistency and transparency in reporting. For further information on Nature Portfolio policies, see our Editorial Policies and the Editorial Policy Checklist.

Statistics

For all statistical analyses, confirm that the following items are present in the figure legend, table legend, main text, or Methods section.

n/a	Confirmed	
☐	☒	The exact sample size (*n*) for each experimental group/condition, given as a discrete number and unit of measurement
☐	☒	A statement on whether measurements were taken from distinct samples or whether the same sample was measured repeatedly
☐	☒	The statistical test(s) used AND whether they are one- or two-sided<br>*Only common tests should be described solely by name; describe more complex techniques in the Methods section.*
☐	☒	A description of all covariates tested
☐	☒	A description of any assumptions or corrections, such as tests of normality and adjustment for multiple comparisons
☐	☒	A full description of the statistical parameters including central tendency (e.g. means) or other basic estimates (e.g. regression coefficient) AND variation (e.g. standard deviation) or associated estimates of uncertainty (e.g. confidence intervals)
☐	☒	For null hypothesis testing, the test statistic (e.g. *F*, *t*, *r*) with confidence intervals, effect sizes, degrees of freedom and *P* value noted<br>*Give P values as exact values whenever suitable.*
☒	☐	For Bayesian analysis, information on the choice of priors and Markov chain Monte Carlo settings
☒	☐	For hierarchical and complex designs, identification of the appropriate level for tests and full reporting of outcomes
☐	☒	Estimates of effect sizes (e.g. Cohen's *d*, Pearson's *r*), indicating how they were calculated

Our web collection on statistics for biologists contains articles on many of the points above.

Software and code

Policy information about availability of computer code

Data collection	Single cell transcriptomes and chromatin landscapes were captured using the 10X Genomics Chromium Single Cell Controller and sequenced using an Illumina NextSeq 500. All in situ and immunofluorescence samples were imaged on a Zeiss LSM 900+Airyscan Microscope using Zeiss ZEN [v3.1 (blue edition)]. Cells were sorted prior to single cell RNA-seq or ATAC-seq using BD FACS S-Aria IIu Cell Sorter 5B 2V 2R or BD FACS 2-Aria IIu Cell Sorter 2B 6V 3R 5YG. Flow cytometry data was collected on the BD Accuri C6 was analyzed using FlowJo [v10.8.0]. Figures were generated using Adobe Illustrator and Photoshop [v2020].
Data analysis	We have used the following software and databases for data analysis : R [3.5.1, 3.6.3], thymusatlastools2 [https://github.com/maehrlab/thymusatlastools2], freezr [https://github.com/ekernf01/freezr], packrat [0.5.0], scanpy [1.6.0], Seurat [2.3.3, 3.2.3], enrichR [3.0], viridis [0.6.1], ArchR [1.0.1], Cis-BP [version 2, http://cisbp.ccbr.utoronto.ca], rGREAT [1.18.0], universalmotif [1.10.2], MACS2 [2.2.7.1], lineup [0.38-3], Phastcon score [http://hgdownload.cse.ucsc.edu/goldenpath/mm10/phastCons60way/mm10.60way.phastCons60wayEuarchontoGlire.bw], magrittr [2.0.1], Matrix [1.3-4], ggplot2 [3.3.5], dplyr [1.0.7], scales [1.1.1], TFBSTools [1.24.0], chromVAR [1.8.0], parallel [3.6.3], RcisTarget [1.6.0], jupyter [1.0.0], metacell [0.3.6], SCENIC [1.2.4], GENIE3 [1.8.0], sna [2.6], network [1.17.1], visNetwork [2.0.9], leiden [0.3.8], igraph [1.2.6], pandas [1.1.4], anndata [0.7.4], seaborn [0.11.1], celloracle [0.6.6], scvelo [0.2.2.dev51+ga7de78a]<br>Custom codes/algorithms are available from https://github.com/maehrlab.

For manuscripts utilizing custom algorithms or software that are central to the research but not yet described in published literature, software must be made available to editors and reviewers. We strongly encourage code deposition in a community repository (e.g. GitHub). See the Nature Portfolio guidelines for submitting code & software for further information.

Data

Policy information about availability of data

All manuscripts must include a data availability statement. This statement should provide the following information, where applicable:

- Accession codes, unique identifiers, or web links for publicly available datasets
- A description of any restrictions on data availability
- For clinical datasets or third party data, please ensure that the statement adheres to our policy

The scRNA-seq and scATAC-seq short reads and count matrices data generated in this study have been deposited in the Gene Expression Omnibus (GEO) database under accession codes GSE182135 [https://www.ncbi.nlm.nih.gov/geo/query/acc.cgi?acc=GSE182135] (scRNA atlas), GSE182136 [https://www.ncbi.nlm.nih.gov/geo/query/acc.cgi?acc=GSE182136] (scRNA knockout experiment) and GSE182134 [https://www.ncbi.nlm.nih.gov/geo/query/acc.cgi?acc=GSE182134] (scATAC atlas).
The motif data used in the scATAC analysis is available in the Cis-BP version 2.00 database for Mus musculus [http://cisbp.ccbr.utoronto.ca/bulk.php]. The conservation scores used in the scATAC analysis are available in the PhastCon score database for the Euarchontoglires clade [http://hgdownload.cse.ucsc.edu/goldenpath/mm10/phastCons60way/mm10.60way.phastCons60wayEuarchontoGlire.bw]

Field-specific reporting

Please select the one below that is the best fit for your research. If you are not sure, read the appropriate sections before making your selection.

☒ Life sciences ☐ Behavioural & social sciences ☐ Ecological, evolutionary & environmental sciences

For a reference copy of the document with all sections, see nature.com/documents/nr-reporting-summary-flat.pdf

Life sciences study design

All studies must disclose on these points even when the disclosure is negative.

Sample size

No statistical methods were used to predetermine sample size. Sample sizes were deemed appropriate because all cell types of a given timepoint were represented in each replicate. Each sample consists of dissected pharyngeal tissue from at least 2 litters, except scRNA E12.5 replicate 2, scATAC E12.5 replicates 1 and 2, and scRNA Foxn1 KO replicate 2 and Foxn1 het replicate 2, each of which was produced from a single litter. From each litter, we pooled the maximum number of embryos of the desired genotype (Pax9VENUS and/or Foxn1nu). All samples consisted of pooled dissected pharyngeal endoderm tissue from multiple embryos, except for scRNA Foxn1 KO replicate 1, which was produced from a single embryo. Approximately 20,000 cells were sorted from each single-cell sample, and all cells were processed through the 10X Chromium Controller to achieve a final library consisting of >3,000 single cell transcriptomes (before data cleaning).
Size by individual sample:
scRNA Pax9VENUS E9.5 replicate 1 - 27 embryos (7 litters)
scRNA Pax9VENUS E9.5 replicate 2 - 21 embryos (5 litters)
scRNA Pax9VENUS E10.5 replicate 1 - 15 embryos (3 litters)
scRNA Pax9VENUS E10.5 replicate 2 - 14 embryos (2 litters)
scRNA Pax9VENUS E10.5 replicate 3 - 12 embryos (5 litters)
scRNA Pax9VENUS E11.5 replicate 1 - 6 embryos (2 litters)
scRNA Pax9VENUS E11.5 replicate 2 - 12 embryos (3 litters)
scRNA Pax9VENUS E11.5 replicate 3 - 16 embryos (4 litters)
scRNA Pax9VENUS E12.5 replicate 1 - 9 embryos (3 litters)
scRNA Pax9VENUS E12.5 replicate 2 - 4 embryos (1 litter)
scATAC Pax9VENUS E11.5 replicate 1 - 32 embryos (9 litters)
scATAC Pax9VENUS E11.5 replicate 2 - 16 embryos (5 litters)
scATAC Pax9VENUS E12.5 replicate 1 - 4 embryos (1 litter)
scATAC Pax9VENUS E12.5 replicate 2 - 4 embryos (1 litter)
scRNA Foxn1 Het E12.5 replicate 1 - 3 embryos (2 litters)
scRNA Foxn1 Het E12.5 replicate 2 - 2 embryos (1 litter)
scRNA Foxn1 KO E12.5 replicate 1 - 3 embryos, littermates to het control (2 litters)
scRNA Foxn1 KO E12.5 replicate 2 - 1 embryo, littermates to het control (1 litter)

Data exclusions

For the scRNA atlas, from the 57,850 cells across 10 samples, cells were processed according to the standard single cell analysis workflow and clustered into groups of similar cell types. Clusters were removed based on the following criteria: Cluster 33 (459 cells) due to high Neurod1 and low Pax9; Cluster 41 (79 cells) due to high Pax3, and low Pax9 and Epcam; Cluster 36 (381 cells) due to high Esam and Pdgfra, moderate Dlx5, and low Pax9 and Epcam; Cluster 38 (247 cells) due to high Pax3 and low Pax9 and Epcam; Cluster 39 (231 cells) due to high Rxrg, Dlx5, and Sox10, and low Pax9; Any cell with over 7% mitochondrial UMIs (2,122 cells); Any cell with under 1% mitochondrial UMIs (270 cells); Any cell with log normalized Hbb-bt expression over 1 (17 cells); leaving 54,044 cells across samples as follows:
E9.5 replicate 1 = 5,734, E9.5 replicate 2 = 7,611
E10.5 replicate 1 = 4,743, E10.5 replicate 2 = 4,129, E10.5 replicate 3 = 4,248
E11.5 replicate 1 = 5,385, E11.5 replicate 2 = 3,141, E11.5 replicate 3 = 7,967
E12.5 replicate 1 = 4,885, E12.5 replicate 2 = 6,201

For the scATAC atlas, we used preestablished exclusion criteria on scATAC-seq data. Namely, cells were removed if they had < 3,000 unique

fragments or < 4 TSS enrichment ratio, and the top N cells (N = number of cells squared per 10,000) per sample with high doublet enrichment were removed. The standard scATAC-seq processing pipeline was followed and cells were clustered and annotated via label transfer based on scRNA-seq pre-filtered cell cluster annotations. Further, clusters having doublet enrichment more than 1 standard deviation away from the average median cluster doublet enrichment were removed and clusters with a high percentage of cells mapping to filtered contaminant scRNAseq populations were removed leaving 10,890 cells across 4 samples as follows:
E11.5 replicate 1 = 3,248, E11.5 replicate 2 = 1,075
E12.5 replicate 1 = 2,813, E12.5 replicate 2 = 3,754

For the scRNA-seq knockout experiment, from the 29,276 cells output by CellRanger, cells were processed according to the standard single cell analysis workflow and clustered into groups of similar cell types. Clusters were removed based on low depth - 3 clusters comprising 3,362 cells, 1,150 cells and 318 cells; and contaminant populations based on label transfer from the prefiltered scRNAseq atlas comprising 207 cells, 132 cells and 37 cells. Additionally any remaining cell with under 2,000 UMIs was removed (2,121 cells), and any remaining cell classified as a contaminant was removed (45 cells) leaving 21,904 cells across 4 samples as follows:
E12.5 Foxn1 heterozygous replicate 1 = 6,805, E12.5 Foxn1 heterozygous replicate 2 = 2,258,
E12.5 Foxn1 knockout replicate 1 = 8,070, E12.5 Foxn1 knockout replicate 2 = 4,771

Replication

All attempts at replication were successful. Correlation across replicates of the same embryonic timepoint demonstrates reproducible data (Supplementary Fig. 1), and visualization of individual replicates by dataset shows strong overlap within timepoints/genotypes (Supplementary Fig. 2, 5, 10). All samples were collected on separate days, except for scRNA E10.5 replicates 1 and 2 and scATAC E12.5 replicates 1 and 2, which consisted of separate litters isolated on the same day, respectively. Samples of Foxn1 KO and heterozygous tissue were collected the same day, but replicates were performed on separate days. The single-cell RNA-seq pharyngeal endoderm dataset comprises two replicates at E9.5, two replicates at E10.5, three replicates at E11.5, and two replicates at E12.5. The single-cell ATAC-seq pharyngeal endoderm dataset is comprises two replicates at E11.5 and two replicates at E12.5. The Foxn1 experiment single-cell RNA-seq dataset comprises two replicates of Foxn1 KO embryos and two replicates of Foxn1 heterozygous embryos, both at E12.5. For RNAscope experiments, two or more biological samples were stained.

Randomization

All single cell analyses were performed in a randomized manner for each of the three datasets, meaning cells from all samples were combined, analyzed and allocated into clusters at a resolution which captured different cell types based on differentially expressed (or accessible in case of scATACseq) markers. Experimental group and sample information was controlled for to overcome batch effects for the analysis of the scRNA datasets. No such batch correction was performed on the scATACseq dataset. For the Foxn1 knockout experiment, heterozygous littermates were used as controls.

Blinding

For the scRNA and scATAC atlas datasets, blinding was not necessary as only one timepoint was collected on a given day. Since embryos of the same genotype were pooled in the Foxn1 scRNA experiments, genotyping was performed prior to tissue processing and thus the experiment could not be performed in a blinded manner. All embryos were processed by a single researcher. All scRNA and scATAC sequencing libraries were prepared and sequenced by a single researcher without blinding. Cell identity, embryonic day, and genotype was blinded during the scRNA and scATAC seq analysis. For the scRNAseq datasets, investigators were not blinded to experimental groups after processing as this would preclude grouping of replicates for analysis. For the scATACseq dataset, investigators were blinded to experimental groups even during the analysis as there was no need to correct for batch effects. Blinding was not required for the in situ hybridization experiments given that all stains were performed on wild type samples. Blinding was not possible for the FACS and flow cytometry experiments because the genotype of each animal was determined prior to tissue processing. Genotyping and tissue processing was performed by a single researcher.

Reporting for specific materials, systems and methods

We require information from authors about some types of materials, experimental systems and methods used in many studies. Here, indicate whether each material, system or method listed is relevant to your study. If you are not sure if a list item applies to your research, read the appropriate section before selecting a response.

Materials & experimental systems

n/a	Involved in the study	
	☒	Antibodies
☒		Eukaryotic cell lines
☒		Palaeontology and archaeology
	☒	Animals and other organisms
☒		Human research participants
☒		Clinical data
☒		Dual use research of concern

Methods

n/a	Involved in the study	
☒		ChIP-seq
	☒	Flow cytometry
☒		MRI-based neuroimaging

Antibodies

Antibodies used

Antibody (Source; Catalog number; clone)
PE/Cy7 anti-mouse Epcam (Biolegend; 118216; G8.8)
anti-Epcam (Biolegend; 118202; G8.8)
anti-Pax9 (Santa Cruz Biotechnology; sc-7746; M-18)
APC anti-mouse CD4 (BD Pharmingen; 553051; RM4-5)
PE anti-mouse CD8a (BD Pharmingen; 553033; 53-6.7)

Validation

Antibodies were utilized according to the manufacturer's recommended usage.

Validation of PE/Cy7 anti-mouse Epcam (Biolegend; 118216; G8.8) and anti-Epcam (Biolegend; 118202; G8.8):
According to the manufacturer, anti-mouse Epcam G8.8 clone reacts to mouse epithelial cells and was demonstrated on TE-71 mouse thymic epithelial cell line by flow cytometry (technical data sheet provided by Biolegend). Epcam expression has been documented in developing mouse endoderm and derivatives using the anti-Epcam G8.8 clone (PMID: 19097184).
Validation of anti-Pax9 (Santa Cruz Biotechnology; sc-7746; M-18):
According to the manufacturer, the anti-Pax9 M-18 clone reacts with mouse Pax9 (technical data sheet provided by Santa Cruz Biotechnology).
Validation of APC anti-mouse CD4 (BD Pharmingen; 553051; RM4-5):
According to the manufacturer, the anti-CD4 RM4-5 clone reacts with thymocytes and subpopulations of mature T lymphocytes (technical data sheet provided by BD Biosceinces). The manufacturer demonstrated specific staining of the anti-CD4 RM4-5 clone on mouse splenocytes via flow cytometry.
Validation of PE anti-mouse CD8a (BD Pharmingen; 553033; 53-6.7):
According to the manufacturer, the anti-CD8a 53-6.7 clone reacts with thymocytes and a subpopulation of mature T lymphocytes (technical data sheet provided by BD Biosciences). The manufacturer demonstrated specific staining of the anti-CD8a 53-6.7 clone on mouse splenocytes via flow cytometry.

Animals and other organisms

Policy information about studies involving animals; ARRIVE guidelines recommended for reporting animal research

Laboratory animals

All mice were housed in an animal facility adhering to intermediate barrier standards, including sterilization or disinfection of all supplies and equipment, usage of PPE (gown, gloves, mask, cap, shoe covers), and air shower entry. The temperature of the facility is maintained between 68-79 Fahrenheit with 30-70% humidity. Mice were exposed to a 12/12 hour light/dark cycle, and were housed socially with no more than 5 mice per cage. Mice had continuous access to standard chow and water.

Strain (Source; Stock number)
C57BL/6J mice (The Jackson Laboratory; 000664): females between the ages of 6 to 8 weeks used for breeding.
Foxn1nu (The Jackson Laboratory; B6.Cg-Foxn1nu/J; 000819): females between the ages of 6 to 8 weeks and males at least 6 weeks of age were used for breeding.

Strain (Source publication)
Pax9VENUS (Stem Cell Res. 2013 Nov;11(3):1003-12)

Wild animals

Wild animals were not used in this study.

Field-collected samples

This study does not include field-collected samples.

Ethics oversight

All mouse experiments were performed in accordance with protocol #2384 approved by the Institutional Animal Care and Use Committee (IACUC) at the University of Massachusetts Medical School.

Note that full information on the approval of the study protocol must also be provided in the manuscript.

Flow Cytometry

Plots

Confirm that:

- [x] The axis labels state the marker and fluorochrome used (e.g. CD4-FITC).
- [x] The axis scales are clearly visible. Include numbers along axes only for bottom left plot of group (a 'group' is an analysis of identical markers).
- [x] All plots are contour plots with outliers or pseudocolor plots.
- [x] A numerical value for number of cells or percentage (with statistics) is provided.

Methodology

Sample preparation

Embryos were dissected at timepoints embryonic day (E)9.5, E10.5, E11.5, and E12.5, where the morning of plug detection was considered E0.5. Dissections were performed in cold RF10-H medium (RPMI 1640 (Gibco; 22400089) supplemented with 2 mM L-glutamine (Gibco; 25030081), 100 IU/mL penicillin and streptomycin (Corning; 352063), and 10% v/v Gibco fetal bovine serum (Gibco; 10437028)). The pharynx region was mechanically dissected from heterozygous Pax9VENUS embryos through removal of the head and lower torso from above the heart. Tissue was washed in cold 1X PBS, then dissociated in 0.05% trypsin-EDTA (GIBCO; 25300-120) and DNase (Sigma; DN25-1g) at 37˚c for 15-20 minutes with P1000 pipette trituration every 5 minutes. Trypsin was deactivated with RF10-H medium. Single cell suspensions were filtered through 40 µm cell strainers (Fisher Scientific; 22363547) and centrifuged at 4˚c, 300 x g for 5 minutes. Cells were treated with 1X RBC lysis buffer (eBioscience; 00-4333-57) at 4˚c for 3 minutes, then washed with FACS buffer (1X PBX + 2% v/v FBS). Cells were stained with a PE/Cy7 anti-mouse CD326 EpCAM antibody (Biolegend; 118216; 1:1000) in FACS buffer at 4˚c for 20 minutes. Dead cells were excluded with 7-AAD (BD Biosciences; 51-68981E).
Thymocytes from 5-week-old littermates (Wildtype, Foxn1nu/wt, and Foxn1nu/wtPax9VENUS/wt) were obtained by mechanical agitation of the thymus. Cells were treated with 1X RBC lysis buffer (eBioscience; 00-4333-57) at 4˚c for 3 minutes, then washed with FACS buffer (1X PBX + 2% v/v FBS). Cells were incubated with anti-mouse CD16/32 (Biolegend; 101302) for 10 mins prior to staining with APC anti-mouse CD4 (BD Pharmingen; 553051) and PE anti-mouse CD8a (BD

	Pharmingen; 553033). Dead cells were excluded with 7-AAD (BD Biosciences; 51-68981E). Data was acquired using a BD Accuri C6.
Instrument	Cell sorting was performed using BD FACS S-Aria IIu Cell Sorter 5B 2V 2R or BD FACS 2-Aria IIu Cell Sorter 2B 6V 3R 5YG.
Software	BD FACSDiva Software was used during FACS, and FlowJo software [v10.8.0] was used to analyze data acquired from FACS.
Cell population abundance	The abundance of sorted cell populations is specified in Supplementary Fig. 1. Cell purity was verified during single cell analysis.
Gating strategy	Gating strategy was applied as follows: dead cell exclusion (7-AAD negative), FSC/SSC, doublet exclusion, fluorescence gates (VENUS, PE/Cy7 double positive). Fluorescence gates were set using an unstained sample as a negative control and single color samples as positive controls.

☒ Tick this box to confirm that a figure exemplifying the gating strategy is provided in the Supplementary Information.
